# Association of breast cancer risk with genetic variants showing differential allelic expression: Identification of a novel breast cancer susceptibility locus at 4q21

**DOI:** 10.18632/oncotarget.12818

**Published:** 2016-10-22

**Authors:** Yosr Hamdi, Penny Soucy, Véronique Adoue, Kyriaki Michailidou, Sander Canisius, Audrey Lemaçon, Arnaud Droit, Irene L Andrulis, Hoda Anton-Culver, Volker Arndt, Caroline Baynes, Carl Blomqvist, Natalia V. Bogdanova, Stig E. Bojesen, Manjeet K. Bolla, Bernardo Bonanni, Anne-Lise Borresen-Dale, Judith S. Brand, Hiltrud Brauch, Hermann Brenner, Annegien Broeks, Barbara Burwinkel, Jenny Chang-Claude, NBCS Collaborators, Fergus J. Couch, Angela Cox, Simon S. Cross, Kamila Czene, Hatef Darabi, Joe Dennis, Peter Devilee, Thilo Dörk, Isabel Dos-Santos-Silva, Mikael Eriksson, Peter A. Fasching, Jonine Figueroa, Henrik Flyger, Montserrat García-Closas, Graham G. Giles, Mark S. Goldberg, Anna González-Neira, Grethe Grenaker-Alnæs, Pascal Guénel, Lothar Haeberle, Christopher A. Haiman, Ute Hamann, Emily Hallberg, Maartje J. Hooning, John L. Hopper, Anna Jakubowska, Michael Jones, Maria Kabisch, Vesa Kataja, Diether Lambrechts, Loic Le Marchand, Annika Lindblom, Jan Lubinski, Arto Mannermaa, Mel Maranian, Sara Margolin, Frederik Marme, Roger L. Milne, Susan L. Neuhausen, Heli Nevanlinna, Patrick Neven, Curtis Olswold, Julian Peto, Dijana Plaseska-Karanfilska, Katri Pylkäs, Paolo Radice, Anja Rudolph, Elinor J. Sawyer, Marjanka K. Schmidt, Xiao-Ou Shu, Melissa C. Southey, Anthony Swerdlow, Rob A.E.M. Tollenaar, Ian Tomlinson, Diana Torres, Thérèse Truong, Celine Vachon, Ans M. W. Van Den Ouweland, Qin Wang, Robert Winqvist, kConFab/AOCS Investigators, Wei Zheng, Javier Benitez, Georgia Chenevix-Trench, Alison M. Dunning, Paul D. P. Pharoah, Vessela Kristensen, Per Hall, Douglas F. Easton, Tomi Pastinen, Silje Nord, Jacques Simard

**Affiliations:** ^1^ Genomics Center, Centre Hospitalier Universitaire de Québec Research Center, Laval University, Quebec, Canada; ^2^ Institut National de la Santé et de la Recherche Médicale U1043, Toulouse, France; ^3^ Centre National de la Recherche Scientifique, Toulouse, France; ^4^ Université de Toulouse, Université Paul Sabatier, Centre de Physiopathologie de Toulouse Purpan, Toulouse, France; ^5^ Centre for Cancer Genetic Epidemiology, Department of Public Health and Primary Care, University of Cambridge, Cambridge, UK; ^6^ Department of Electron Microscopy/Molecular Pathology, The Cyprus Institute of Neurology and Genetics, Nicosia, Cyprus; ^7^ Netherlands Cancer Institute, Antoni van Leeuwenhoek hospital, Amsterdam, The Netherlands; ^8^ Centre de Recherche du CHU de Québec – Université Laval, Faculté de Médecine, Département de Médecine Moléculaire, Université Laval, Quebec, Canada; ^9^ Lunenfeld-Tanenbaum Research Institute of Mount Sinai Hospital, Toronto, Canada; ^10^ Department of Molecular Genetics, University of Toronto, Toronto, Canada; ^11^ Department of Epidemiology, University of California Irvine, Irvine, CA, USA; ^12^ Division of Clinical Epidemiology and Aging Research, German Cancer Research Center, Heidelberg, Germany; ^13^ Centre for Cancer Genetic Epidemiology, Department of Oncology, University of Cambridge, Cambridge, UK; ^14^ Department of Oncology, Helsinki University Hospital, University of Helsinki, Helsinki, Finland; ^15^ Department of Radiation Oncology, Hannover Medical School, Hannover, Germany; ^16^ Gynaecology Research Unit, Hannover Medical School, Hannover, Germany; ^17^ Copenhagen General Population Study, Herlevand Gentofte Hospital, Copenhagen University Hospital, Herlev, Denmark; ^18^ Department of Clinical Biochemistry, Herlev and Gentofte Hospital, Copenhagen University Hospital, Herlev, Denmark; ^19^ Faculty of Health and Medical Sciences, University of Copenhagen, Copenhagen, Denmark; ^20^ Division of Cancer Prevention and Genetics, Istituto Europeo di Oncologia, Milan, Italy; ^21^ Department of Cancer Genetics, Institute for Cancer Research, Oslo University Hospital Radiumhospitalet, Oslo, Norway; ^22^ Department of Medical Epidemiology and Biostatistics, Karolinska Institutet, Stockholm, Sweden; ^23^ Dr. Margarete Fischer-Bosch-Institute of Clinical Pharmacology, Stuttgart, Germany; ^24^ University of Tübingen, Tübingen, Germany; ^25^ German Cancer Consortium, German Cancer Research Center, Heidelberg, Germany; ^26^ Division of Preventive Oncology, German Cancer Research Center and National Center for Tumor Diseases, Heidelberg, Germany; ^27^ Department of Obstetrics and Gynecology, University of Heidelberg, Heidelberg, Germany; ^28^ Molecular Epidemiology Group, German Cancer Research Center, Heidelberg, Germany; ^29^ Division of Cancer Epidemiology, German Cancer Research Center, Heidelberg, Germany; ^30^ University Cancer Center Hamburg, University Medical Center Hamburg-Eppendorf, Hamburg, Germany; ^31^ Department of Oncology, Haukeland University Hospital, Bergen, Norway; ^32^ Section of Oncology, Institute of Medicine, University of Bergen, Bergen, Norway; ^33^ Department of Pathology, Akershus University Hospital, Lørenskog, Norway; ^34^ Department of Breast-Endocrine Surgery, Akershus University Hospital, Lørenskog, Norway; ^35^ Department of Breast and Endocrine Surgery, Oslo University Hospital, Ullevål, Oslo, Norway; ^36^ Department of Research, Vestre Viken, Drammen, Norway; ^37^ Institute of Clinical Medicine, Faculty of Medicine, University of Oslo, Oslo, Norway; ^38^ National Advisory Unit on Late Effects after Cancer Treatment, Oslo University Hospital Radiumhospitalet, Oslo, Norway; ^39^ Department of Oncology, Oslo University Hospital Radiumhospitalet, Oslo, Norway; ^40^ Department of Radiology and Nuclear Medicine, Oslo University Hospital Radiumhospitalet, Oslo, Norway; ^41^ Oslo University Hospital, Oslo, Norway; ^42^ Department of Laboratory Medicine and Pathology, Mayo Clinic, Rochester, MN, USA; ^43^ Sheffield Cancer Research, Department of Oncology and Metabolism, University of Sheffield, Sheffield, UK; ^44^ Academic Unit of Pathology, Department of Neuroscience, University of Sheffield, Sheffield, UK; ^45^ Department of Pathology, Leiden University Medical Center, Leiden, The Netherlands; ^46^ Department of Human Genetics, Leiden University Medical Center, Leiden, The Netherlands; ^47^ Department of Non-Communicable Disease Epidemiology, London School of Hygiene and Tropical Medicine, London, UK; ^48^ Department of Gynaecology and Obstetrics, University Hospital Erlangen, Friedrich-Alexander University Erlangen-Nuremberg, Comprehensive Cancer Center Erlangen-EMN, Erlangen, Germany; ^49^ David Geffen School of Medicine, Department of Medicine Division of Hematology and Oncology, University of California at Los Angeles, Los Angeles, CA, USA; ^50^ Usher Institute of Population Health Sciences and Informatics, The University of Edinburgh Medical School, Edinburgh, UK; ^51^ Division of Cancer Epidemiology and Genetics, National Cancer Institute, Rockville, MD, USA; ^52^ Department of Breast Surgery, Herlev and Gentofte Hospital, Copenhagen University Hospital, Herlev, Denmark; ^53^ Cancer Epidemiology Centre, Cancer Council Victoria, Melbourne, Australia; ^54^ Centre for Epidemiology and Biostatistics, Melbourne School of Population and Global Health, The University of Melbourne, Melbourne, Australia; ^55^ Department of Medicine, McGill University, Montreal, Canada; ^56^ Division of Clinical Epidemiology, Royal Victoria Hospital, McGill University, Montreal, Canada; ^57^ Human Cancer Genetics Program, Spanish National Cancer Research Centre, Madrid, Spain; ^58^ Cancer & Environment Group, Center for Research in Epidemiology and Population Health (CESP), INSERM, University Paris-Sud, University Paris-Saclay, VilleJuif, France; ^59^ Department of Preventive Medicine, Keck School of Medicine, University of Southern California, Los Angeles, CA, USA; ^60^ Molecular Genetics of Breast Cancer, German Cancer Research Center, Heidelberg, Germany; ^61^ Department of Health Sciences Research, Mayo Clinic, Rochester, MN, USA; ^62^ Department of Medical Oncology, Family Cancer Clinic, Erasmus MC Cancer Institute, Rotterdam, The Netherlands; ^63^ Department of Genetics and Pathology, Pomeranian Medical University, Szczecin, Poland; ^64^ Division of Genetics and Epidemiology, the Institute of Cancer Research, London, UK; ^65^ Cancer Center of Eastern Finland, University of Eastern Finland, Kuopio, Finland; ^66^ Central Finland Hospital District, Jyväskylä Central Hospital, Jyväskylä, Finland; ^67^ Vesalius Research Center, Leuven, Belgium; ^68^ Laboratory for Translational Genetics, Department of Oncology, University of Leuven, Leuven, Belgium; ^69^ University of Hawaii Cancer Center, Honolulu, HI, USA; ^70^ Department of Molecular Medicine and Surgery, Karolinska Institutet, Stockholm, Sweden; ^71^ Institute of Clinical Medicine, Pathology and Forensic Medicine, University of Eastern Finland, Kuopio, Finland; ^72^ Imaging Center, Department of Clinical Pathology, Kuopio University Hospital, Kuopio, Finland; ^73^ Department of Oncology - Pathology, Karolinska Institutet, Stockholm, Sweden; ^74^ National Center for Tumor Diseases, University of Heidelberg, Heidelberg, Germany; ^75^ Department of Population Sciences, Beckman Research Institute of City of Hope, Duarte, CA, USA; ^76^ Department of Obstetrics and Gynecology, Helsinki University Hospital, University of Helsinki, Helsinki, Finland; ^77^ Multidisciplinary Breast Center, Department of Oncology, University Hospitals Leuven, Leuven, Belgium; ^78^ Research Center for Genetic Engineering and Biotechnology “Georgi D. Efremov”, Macedonian Academy of Sciences and Arts, Skopje, Republic of Macedonia; ^79^ Laboratory of Cancer Genetics and Tumor Biology, Cancer and Translational Medicine Research Unit, Biocenter Oulu, University of Oulu, Oulu, Finland; ^80^ Laboratory of Cancer Genetics and Tumor Biology, Northern Finland Laboratory Centre Oulu, Oulu, Finland; ^81^ Unit of Molecular Bases of Genetic Risk and Genetic Testing, Department of Preventive and Predictive Medicine, Fondazione Istituto Di Ricovero e Cura a Carattere, Scientifico, Istituto Nazionale Tumori, Milan, Italy; ^82^ Research Oncology, Guy's Hospital, King's College London, London, UK; ^83^ Division of Epidemiology, Department of Medicine, Vanderbilt-Ingram Cancer Center, Vanderbilt University School of Medicine, Nashville, TN, USA; ^84^ Department of Pathology, The University of Melbourne, Melbourne, Australia; ^85^ Division of Genetics and Epidemiology & Division of Breast Cancer Research, The Institute of Cancer Research, London, UK; ^86^ Department of Surgery, Leiden University Medical Center, Leiden, The Netherlands; ^87^ Wellcome Trust Centre for Human Genetics and Oxford NIHR Biomedical Research Centre, University of Oxford, Oxford, UK; ^88^ Institute of Human Genetics, Pontificia Universidad Javeriana, Bogota, Colombia; ^89^ Department of Clinical Genetics, Erasmus University Medical Center, Rotterdam, The Netherlands; ^90^ Peter MacCallum Cancer Center, the University of Melbourne, Melbourne, Australia; ^91^ Centro de Investigación en Red de Enfermedades Raras, Valencia, Spain; ^92^ Department of Genetics, QIMR Berghofer Medical Research Institute, Brisbane, Australia; ^93^ Department of Clinical Molecular Biology, Oslo University Hospital, University of Oslo, Oslo, Norway; ^94^ Department of Human Genetics, McGill University, Montreal, Quebec, Canada; ^95^ McGill University and Genome Quebec Innovation Centre, Montreal, Quebec, Canada

**Keywords:** breast cancer, genetic susceptibility, association studies, differential allelic expression, cis-regulatory variants

## Abstract

There are significant inter-individual differences in the levels of gene expression. Through modulation of gene expression, *cis*-acting variants represent an important source of phenotypic variation. Consequently, c*is*-regulatory SNPs associated with differential allelic expression are functional candidates for further investigation as disease-causing variants. To investigate whether common variants associated with differential allelic expression were involved in breast cancer susceptibility, a list of genes was established on the basis of their involvement in cancer related pathways and/or mechanisms. Thereafter, using data from a genome-wide map of allelic expression associated SNPs, 313 genetic variants were selected and their association with breast cancer risk was then evaluated in 46,451 breast cancer cases and 42,599 controls of European ancestry ascertained from 41 studies participating in the Breast Cancer Association Consortium. The associations were evaluated with overall breast cancer risk and with estrogen receptor negative and positive disease. One novel breast cancer susceptibility locus on 4q21 (rs11099601) was identified (OR = 1.05, *P* = 5.6x10^-6^). rs11099601 lies in a 135 kb linkage disequilibrium block containing several genes, including, *HELQ*, encoding the protein HEL308 a DNA dependant ATPase and DNA Helicase involved in DNA repair, *MRPS18C* encoding the Mitochondrial Ribosomal Protein S18C and *FAM175A (ABRAXAS)*, encoding a *BRCA1* BRCT domain-interacting protein involved in DNA damage response and double-strand break (DSB) repair. Expression QTL analysis in breast cancer tissue showed rs11099601 to be associated with *HELQ* (*P* = 8.28x10^-14^), *MRPS18C* (*P* = 1.94x10^-27^) and *FAM175A* (*P* = 3.83x10^-3^), explaining about 20%, 14% and 1%, respectively of the variance inexpression of these genes in breast carcinomas.

## INTRODUCTION

Breast cancer is a complex disease with a strong heritable component. Great efforts have been made during the last decades to elucidate the underlying etiology of this disease. Three classes of breast cancer susceptibility alleles with different levels of risk and prevalence in the population are now recognized. High-risk alleles such as *BRCA1* [1, 2], *BRCA2* [3, 4] and *TP53* [5] explain approximately 20% of the inherited susceptibility, intermediate-risk alleles in DNA-repair genes increase this proportion by ~5% [6-18], and common lower-risk alleles, of which approximately 100 have been identified to date through genome-wide association studies (GWAS), replication and custom genotyping efforts, explain approximately 16% of the risk [19-41]. Recent evidence suggests that a substantial fraction of the residual aggregation could be explicable by other common variants not yet identified [35, 40].

Global analysis of genome-wide association study (GWAS) data has shown that the large majority of common variants associated with susceptibility to cancer lie in non-coding regions, and are presumed to mediate risk through regulation of gene expression [42, 43]. Indeed, variations in gene expression occur commonly in the human genome, playing a key role in human phenotypic variability [44-46]. Studies of allelic imbalances in expression indicate that allele-specific differences among transcripts within an individual can affect up to 30% of loci and, at the population level, ~30% of expressed genes show evidence of *cis*-regulation by common polymorphic alleles [47]. Recent evidence has also suggested that differences in gene expression play a critical role in the underlying phenotypic variation associated with many complex genetic diseases [48]. A recent report performed expression quantitative trait loci (*cis*-eQTL) analyses for mRNA expression in five tumor types (breast, colon, kidney, lung and prostate) and tested 149 known cancer risk loci for eQTL effects [49]. They observed that 42 of these risk loci were significantly associated with eQTLs in at least one gene within 500 kb, eight of which were breast cancer risk loci [49]. Furthermore, a recent study has shown that close to half of the known risk alleles for estrogen receptor (ER)-positive breast cancer are eQTLs acting upon major determinants of gene expression in tumors [50]. These results suggest that additional cancer susceptibility loci may be identified through studying genetic variants affecting regulation of gene expression.

In the current study, we performed a breast cancer association study of 313 genetic variants showing evidence of association with differential allelic expression (DAE) selected from 175 genes involved in cancer etiology. These included genes involved in DNA repair (homologous recombination (HR) and DNA interstrand crosslink (ICL) repair), interacting and/or modulating BRCA1 and BRCA2 cellular functions, cell cycle control, centrosome amplification and AURKA interactions, apoptosis, ubiquitination, known tumor suppressors and mitotic and other kinases, as well as sex steroid action and mammographic density. We used genotype data derived from the iCOGS (Collaborative Oncological Gene-environment Study) custom array [35] to investigate the role of these variants on breast cancer risk.

## RESULTS

### Overall and subtype-specific breast cancer risk association analyses

For the one hundred seventy-five selected genes involved in cancer-related pathways, we identified a set of 355 genetic variants showing evidence of association with DAE (see S1 Table for complete list of genes and SNPs). Of the 355 SNPs originally selected, 313 (representing 227 independent SNPs with pairwise r^2^ < 0.1) were successfully genotyped. Thirty-two variants were excluded because of low Illumina design scores, and eleven SNPs were excluded because of low call-rates and/or evidence of deviation from Hardy Weinberg Equilibrium (*P*-value < 10^-7^), respectively. Eighty-two SNPs were originally submitted to be included on the iCOGS array but were replaced with surrogates in the final design of the array. Association results with breast cancer risk for all 313 SNPs are presented in S2 Table.

Thirteen SNPs from ten different loci were associated with overall breast cancer risk (*P* < 10^-2^) (Table 1). Of these, three SNPs, namely rs11099601, rs656040 and rs738200, had associations with an increased overall risk of breast cancer that reached *P* < 10^-4^ (approximate significance cut-off after Bonferroni correction, given 313 tests). No significant evidence of heterogeneity was observed among odds ratios (ORs) for these SNPs among studies (*I^2^* and *P*-values are given in S1 Figure). The minor alleles of rs11099601 at 4q21 (OR = 1.05, *P* = 5.6x10^-6^), rs656040 at 11q13 (OR = 1.05, *P* = 1.52 x10^-5^), and rs738200 at 22q12.1 (OR = 1.09, *P* = 5.32x10^-5^) were associated with increased overall risk of the disease. rs11099601 was associated with both ER-positive (*P* = 5.22x10^-6^) and ER-negative (*P* = 4.08x10^-4^) breast cancer risk (*P* for difference 0.93) while rs656040 and rs738200 appeared primarily associated with ER-positive disease (*P* = 5.96x10^-5^ and *P* = 7.21x10^-6^, respectively), although the difference between ER-positive and ER-negative disease was not statistically significant for these two latter SNPs (*P* for difference 0.096 and 0.242, respectively). Of these three SNPs, only variant rs110099601 represents a novel low penetrance breast cancer susceptibility locus. The two other variants, (rs656040 at 11q13 and rs738200 at 22q12.1) which were not known to be associated with breast cancer risk at the time the current study was designed, were identified through the main analyses of the iCOGS array. rs656040 is located on 11q13 in the 3’-UTR region of the *SNX32* gene, approximately 6.8Kb upstream of *MUS81*, and is associated with differential allelic expression of this latter gene (S2 Figure). rs656040 is partially correlated with rs3903072 (r^2^ = 0.38), which was previously identified as associated with breast cancer risk at *P* < 10^-8^ in the combined GWAS and iCOGS analysis reported in Michailidou et al. [35]. Similarly, variant rs738200, located on locus 22q12 in the tetratricopeptide repeat domain 28 gene (*TTC28*), falls within a 610 kb interval (Build 37 coordinates chr22: 28,314,612-28,928,858) on chromosome 22 recently shown to be associated with breast cancer risk (smallest *P* = 8.2×10^−22^, for rs62237573). This interval lies approximately 100 kb centromeric to *CHEK2*, and further analysis showed that the associated SNPs were correlated with the deleterious *CHEK2* variant c.1100delC and adjustment for this variant suggested the signal is driven by *CHEK2* c.1100delC [40]. rs738200 was genotyped as a surrogate to our originally selected SNP for this locus (rs9620797), and therefore no allelic expression data were available for this SNP.

**Table 1:** Associations with breast cancer risk for SNPs showing evidence of differential allelic expression (overall *p* <0.01)

SNP	Chr^a^	Position^b^	Alleles^c^	MAF^d^	OR^e^_ overall risk (95%CI)	P1df^f^_overall risk	OR^e^ ER+ (95% Cl)	P1df^f^_ER+	OR^e^_ER-(95% Cl)	P1df^f^_ER-	Genes
rs697004	1	211842808	G/T	0.32	0.96 (0.94-0.98)	3.67 × 10^−04^	0.96 (0.94-0.99)	2.84 × 10^−03^	0.97 (0.93-1.01)	1.46 × 10^−01^	*NEK2*
rs13447450	1	91965850	C/T	0.37	0.97 (0.95-0.99)	1.16 × 10^−03^	0.96 (0.94-0.99)	1.53 × 10^−03^	0.96 (0.92-1.00)	3.61 × 10^−02^	*CDC7*
rs12125947	1	91990487	T/C	0.49	0.97 (0.95-0.99)	1.59 × 10^−03^	0.97 (0.95-0.99)	4.23 × 10^−03^	0.97 (0.93-1.01)	9.47 × 10^−02^	*CDC7*
rs10490250	2	58509628	A/C	0.21	1.03 (1.01-1.06)	7.51 × 10^−03^	1.03 (1.01-1.06)	1.85 × 10^−02^	1.05 (1.01-1.10)	4.29 × 10^−02^	*FANCL*
rs13099560	3	48204768	C/T	0.34	0.97 (0.95-0.99)	3.99 × 10^−03^	0.96 (0.94-0.98)	8.75 × 10^−04^	1.00 (0.96-1.04)	9.24 × 10^−01^	*CDC25A*
**rs11099601**	4	84382763	A/G	0.50	1.05 (1.03-1.07)	**5.62 × 10^−06^**	1.05 (1.03-1.08)	**5.22 × 10^−06^**	1.07 (1.03-1.11)	4.08 × 10^−04^	*HELQ, MRPS18C, FAM175A*
rs17355027	4	84388915	C/T	0.08	0.95 (0.92-0.98)	4.46 × 10^−03^	0.95 (0.91-0.99)	1.17 × 10^−02^	0.92 (0.86-0.98)	1.55 × 10^−02^	*FAM175A*
rs2362974	5	36156654	C/T	0.12	0.96 (0.93-0.99)	5.96 × 10^−03^	0.97 (0.93-1.00)	5.45 × 10^−02^	0.99 (0.94-1.06)	9.56 × 10^−01^	*SKP2*
rs733590	6	36645203	T/C	0.36	1.04 (1.03-1.06)	1.77 × 10^−04^	1.03 (1.01-1.06)	7.30 × 10^−03^	1.05 (1.01-1.09)	1.99 × 10^−02^	*CDKN1A*
**rs656040**	11	65621057	C/T	0.33	1.05 (1.02-1.07)	**1.52 × 10^−05^**	1.05 (1.03-1.07)	**5.96 × 10^−05^**	1.03 (0.99-1.07)	2.25 × 10^−01^	*SNX32, CFL1, MUS81*
rs570933	15	43824030	T/C	0.29	0.97 (0.95-0.99)	7.18 × 10^−03^	0.97 (0.95-0.99)	1.78 × 10^−02^	0.96 (0.92-1.00)	2.89 × 10^−02^	*TP53BP1, MAP1A, HISPPD2A*
rs7234479	18	20599564	A/C	0.11	1.05 (1.02-1.08)	1.59 × 10^−03^	1.05 (1.01-1.09)	7.57 × 10^−03^	1.04 (0.98-1.10)	1.52 × 10^−01^	*RBBP8*
**rs738200**	22	28792887	C/T	0.10	1.07 (1.03-1.10)	**5.32 × 10^−05^**	1.09 (1.05-1.13)	**7.21 × 10^−06^**	1.03 (0.97-1.09)	3.87 × 10^−01^	*TTC28, CHEK2*

a Chromosome

b Build 37 position

c Major/minor allele, based on the forward strand and minor allele frequency in Europeans

d Mean minor allele frequency over all European controls in iCOGS

e Per-allele OR for the minor allele relative to the major allele

f One-degree-of-freedom P-value

SNPs highlighted in bold are those with associations for overall breast cancer risk reaching p<10-4 (significance cut-off after Bonferroni correction)

All variants associated with overall breast cancer risk with *P* < 10^-2^ included in Table 1 were also evaluated for association with breast cancer risk in *BRCA1* and *BRCA2* mutation carriers within the Consortium of Investigators of Modifiers of *BRCA1* and *BRCA2* (CIMBA) in a total of 15 252 *BRCA1* and 8 211 *BRCA2* carriers. However, none of the SNPs showed associations with breast cancer risk, including rs11099601, which had a *P*-value of 0.89 and 0.78 in *BRCA1* and *BRCA2* carriers respectively.

**Figure 1:**
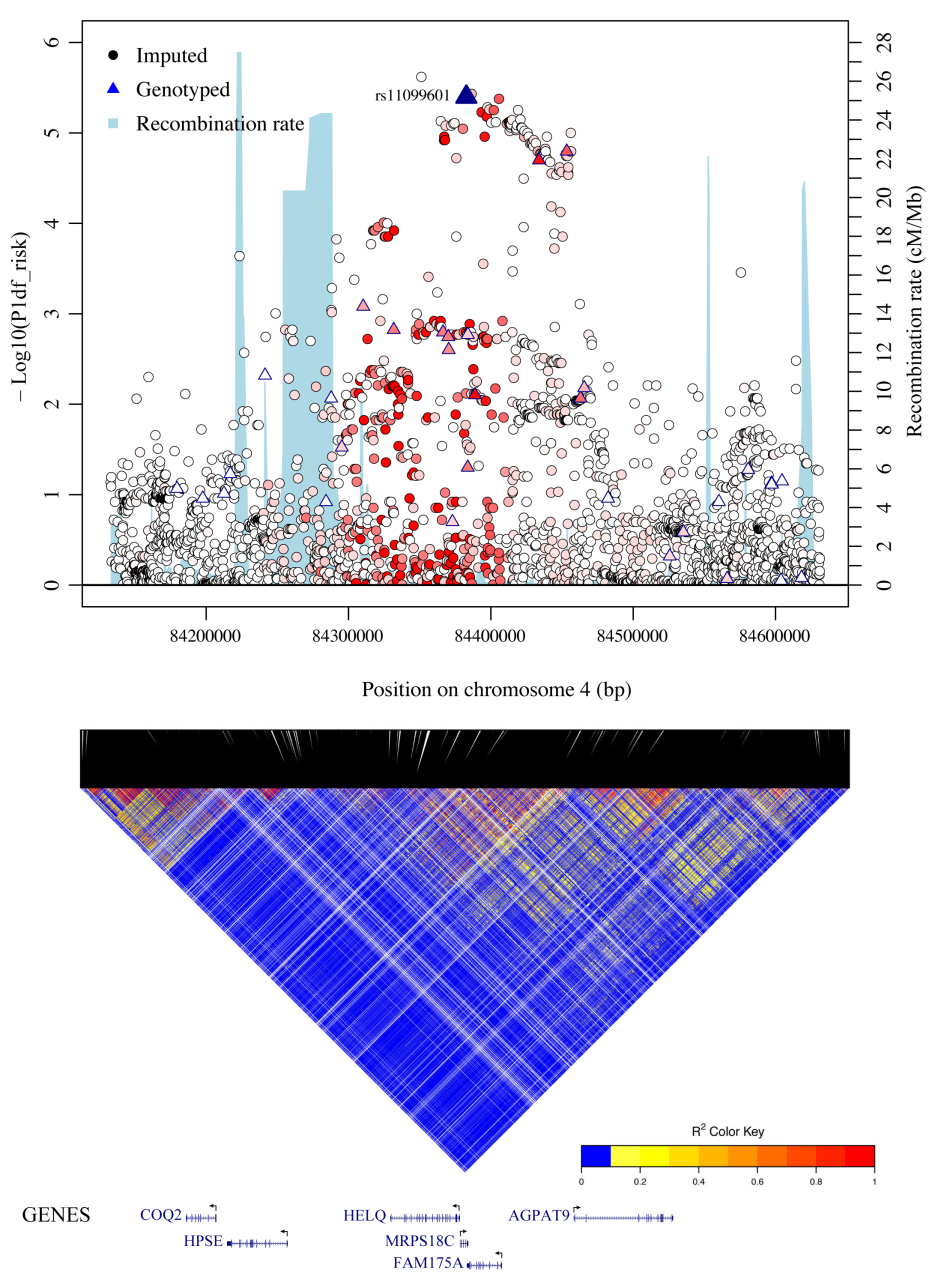
Regional plots of breast cancer risk association at 4q21 Regional plot of association result, recombination hotspots and LD for the 4q21: 84,132,874-84,631,193 loci. The index SNP rs11099601 is plotted as a blue triangle. Directly genotyped SNPs are represented as triangles and imputed SNPs (r^2^ > 0.3, MAF > 0.02) are represented as circles. The LD (r^2^) for the index SNP with each SNP was computed based on European ancestry subjects included in the 1000 Genome Mar 2012 EUR. Pairwise r^2^ values are plotted using a red scale, where white and red signify r^2^ = 0 and 1, respectively. *P*-values were from the single-marker analysis based on logistic regression models after adjusted for age, study sites and the first six principal components plus one additional principal component for the LMBC in analyses of data from European descendants. SNPs are plotted according to their chromosomal position: physical locations are based on GRCh37/hg19. Gene annotation was based on the NCBI RefSeq genes from the UCSC Genome Browser.

rs11099601 lies on 4q21 in a region containing numerous genes including *FAM175A* (*ABRAXAS)*, *HELQ* and *MRPS18C*. It was selected on the basis of its association with differential allelic expression in *FAM175A* (see S2 Figure). In order to further map the novel association at this locus, we imputed genotype data for 2,456 common variants across a 500 kb region centered on rs11099601 (chr4: 84,132,874-84,631,193 from GRCh37/hg19) using the March 2012 release of the 1000 Genomes Project as a reference panel. Subsequent association analysis for overall breast cancer risk revealed that rs11099601 was located in a region of approximately 135 kb exhibiting strong LD (Figure 1). SNP rs11099601 remained one of the most strongly associated SNPs, along with three other perfectly correlated imputed SNPs (r^2^ = 1.0), namely rs4235062 (*P* = 2.40x10^-6^), rs6838225 (*P* = 3.70x10^-6^) and rs13142756 (*P* = 4x10^-6^) (Figure 1) (S3 Table). 88 SNPs were strongly correlated with rs11099601 (r^2^ > 0.8; S4 Table) and hence not distinguishable as potential causal variants on the basis of association data alone.

### Functional annotation of locus 4q21

In order to identify potential candidate causal variants at the 4q21 locus, we overlaid the associated variants with publicly available functional annotations. The analysis was performed on the subset of 88 variants strongly correlated with the lead SNP, rs11099601 (r^2^ > 0.8). We first performed analyses using RegulomeDB (http://www.regulomedb.org) in order to obtain a predicted score of functionality for the set of variants. Interestingly, variant rs11099601 was one of three variants with the highest scores, along with rs1494961 and rs6535481. The corresponding RegulomeDB score (1f) (S4 Table) suggests that these variants are likely to affect transcription factor binding and to be linked to expression of a target gene. The scores for the other three strongest associated SNPs, namely rs4235062, rs6838225 and rs13142756, were not suggestive of functionality (S4 Table - for a description of the RegulomeDB scoring scheme and referenced datatypes refer to http://www.regulomedb.org). Five other highly correlated SNPs (rs10008742, rs6844460, rs7691492, rs526064, rs813298), however, also had high scores (2b), albeit lower than that of the lead SNP rs11099601, indicative of likely affecting transcription factor binding.

**Figure 2:**
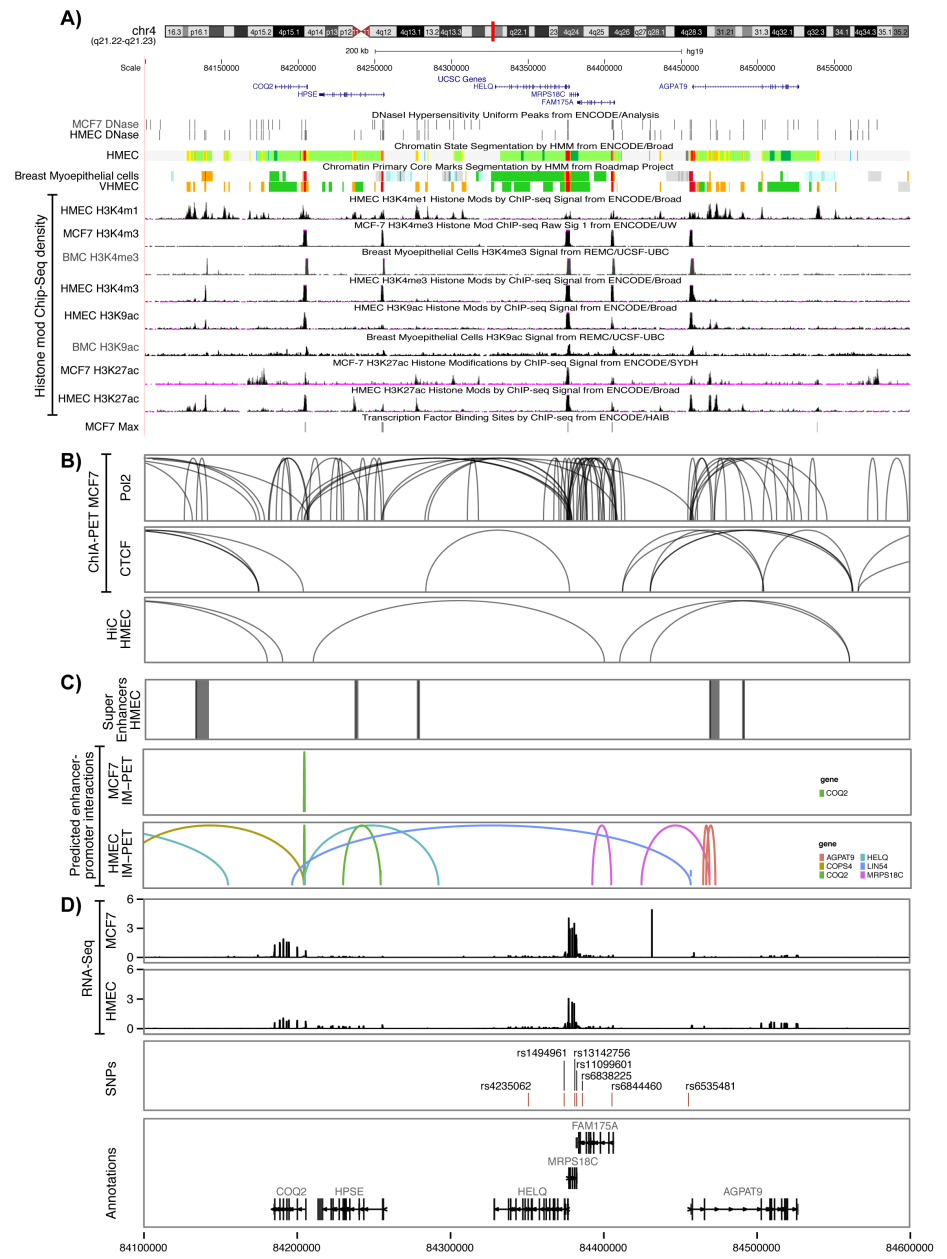
Functional annotation of the 4q21 locus **A.** Functional annotations using data from the ENCODE and NIH Roadmap Epigenomics projects. From top to bottom, epigenetic signals evaluated included DNase clusters in MCF7 and HMEC cells, chromatin state segmentation by Hidden Markov Model (ChromHMM) in HMEC, breast myoepithelial cells (BMC) and Variant human mammary epithelial cells (vHMEC), where red represents an active promoter region, orange a strong enhancer and yellow a poised enhancer respectively (the detailed color scheme of chromatin states is described in the UCSC browser), histone modifications in MCF7, HMEC and BMC cell lines; and overlap between candidate variants and Max binding site in MCF7 cells. All tracks were generated by the UCSC genome browser (hg 19). **B.** Long-range chromatin interactions. From top to bottom, ChIA-Pet interactions for PolII and CTCF in MCF7 cells and Hi-C interactions in HMEC cells. The ChIA-PET raw data available on GEO under the following accession (GSE63525.K56, GSE33664, GSE39495) were processed with the GenomicRanges package. **C.** Maps of mammary cell super-enhancer locations as defined in Hnisz et al. are shown in HMEC cells. Predicted enhancer-promoter determined interactions in MCF7 and HMEC cells, as defined by the integrated method for predicting enhancer targets (IM-PET) are shown. **D.** RNA-Seq data from MCF7 and HMEC cell lines. The value of the RNA-Seq analysis corresponds to the mean RPM value for *FAM175A*, *MRPS18C*, *HELQ*, *AGPAT9*, *HSPE* and *COQ2* from four HMEC and 19 MCF7 datasets, respectively. The annotation was obtained through the Bioconductor annotation package TxDb.Hsapiens.UCSC.hg19.knownGene. The tracks have been generated using ggplot2 and ggbio library in R.

We then analysed ENCODE chromatin biofeatures, namely DNase I hypersensitivity, chromatin state segmentation by HMM (chromHMM) and histone modifications of epigenetic markers H3K4, H3K9 and H3K27 in all breast cell lines available in ENCODE, including breast myoepithelial cells, HMEC mammary cell line, and breast cancer cell line MCF-7. Analysis of these biofeatures revealed an overlap between H3K9Ac, a histone mark associated with active promoters, and our candidate variant, rs11099601 in breast myoepithelial cells. Further analysis of other genotyped and imputed variants correlated with rs11099601, revealed that only rs6844460 (*P* = 4.2x10^-6^, r^2^ = 0.967) overlapped with several chromatin biofeatures in mammary cells. rs6844460, which is located within intron 1 of *FAM175A*, overlapped with a DNase hypersensitivity site in MCF-7 cells, with H3K4me3 histone marks (associated with active promoters) in breast myoepithelial cells, HMEC and MCF-7 cell lines, with H3K9Ac histone marks in both breast myoepithelial cells and HMEC cells, and with H3K27Ac histone marks in HMEC. ChromHMM data also predicts that this variant lies within an active promoter region in breast cell lines (Figure 2A). Moreover, rs6844460 overlapped with a binding site for transcription factor Max (MYC Associated Factor X) in MCF7 cells.

In order to identify potential target genes, we analysed enhancer-promoter interactions using ChiA-PET data for CCCTC-binding factor (CTCF) and DNA polymerase II (PolII) in MCF-7 breast tumour derived cells. Multiple, dense, chromosomal interactions were observed in ChiA-PET data for PolII across most of the entire locus, especially in the region encompassing rs11099601, in the vicinity of the promoter regions of *HELQ, MRPS18C* and *FAM175A* genes. ChiA-PET data for CTCF in MCF-7 cells showed fewer interactions, none of which encompassed variant rs11099601. Similarly Hi-C data revealed few interactions in HMEC cells, none of which included our top candidate SNP (Figure 2B).

Lastly, although super-enhancers mapped to the 4q21 locus in HMEC mammary cells, none overlapped with our top candidate SNPs (Figure 2C). Predicted enhancer-promoter interactions were observed with the promoters of *AGPAT9*, *COQ2*, *HELQ* and *MRPS18C* genes in HMEC cells. However amongst these, only interactions with *MRPS18C* overlapped with our top putative candidate functional variants (rs11099601 and rs6844460) (Figure 2C).

**Figure 3:**
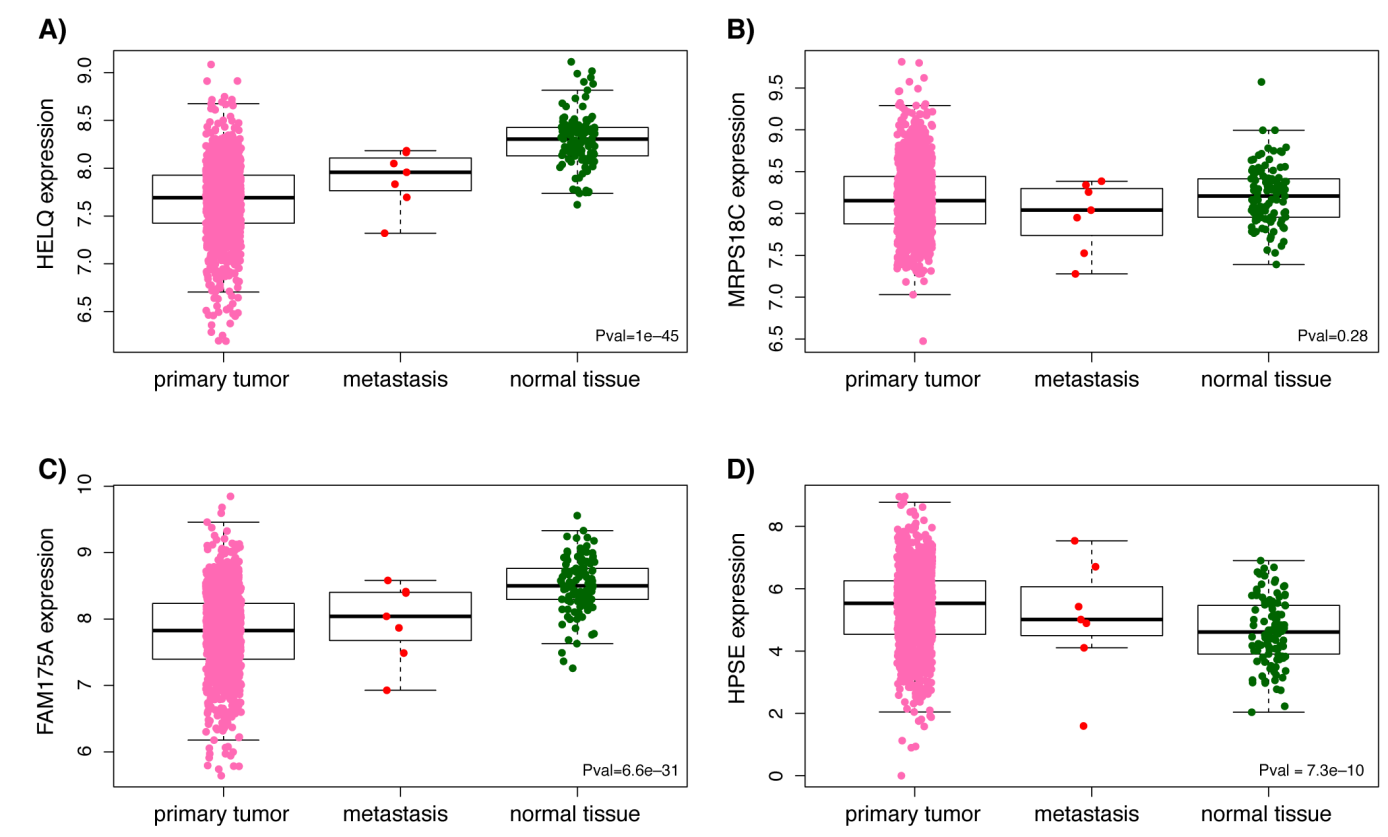
Boxplots representing differential expression of *HELQ (A), MRPS18C (B), FAM175A (C)* and *HPSE (D)* in breast tissues Differential expression between normal breast and tumor tissue was determined by a Kruskal-Wallis rank sum test using TCGA breast cancer RNAseq data from primary tumor, metastasis and adjacent normal. Horizontal bars indicate mean expression levels.

**Figure 4:**
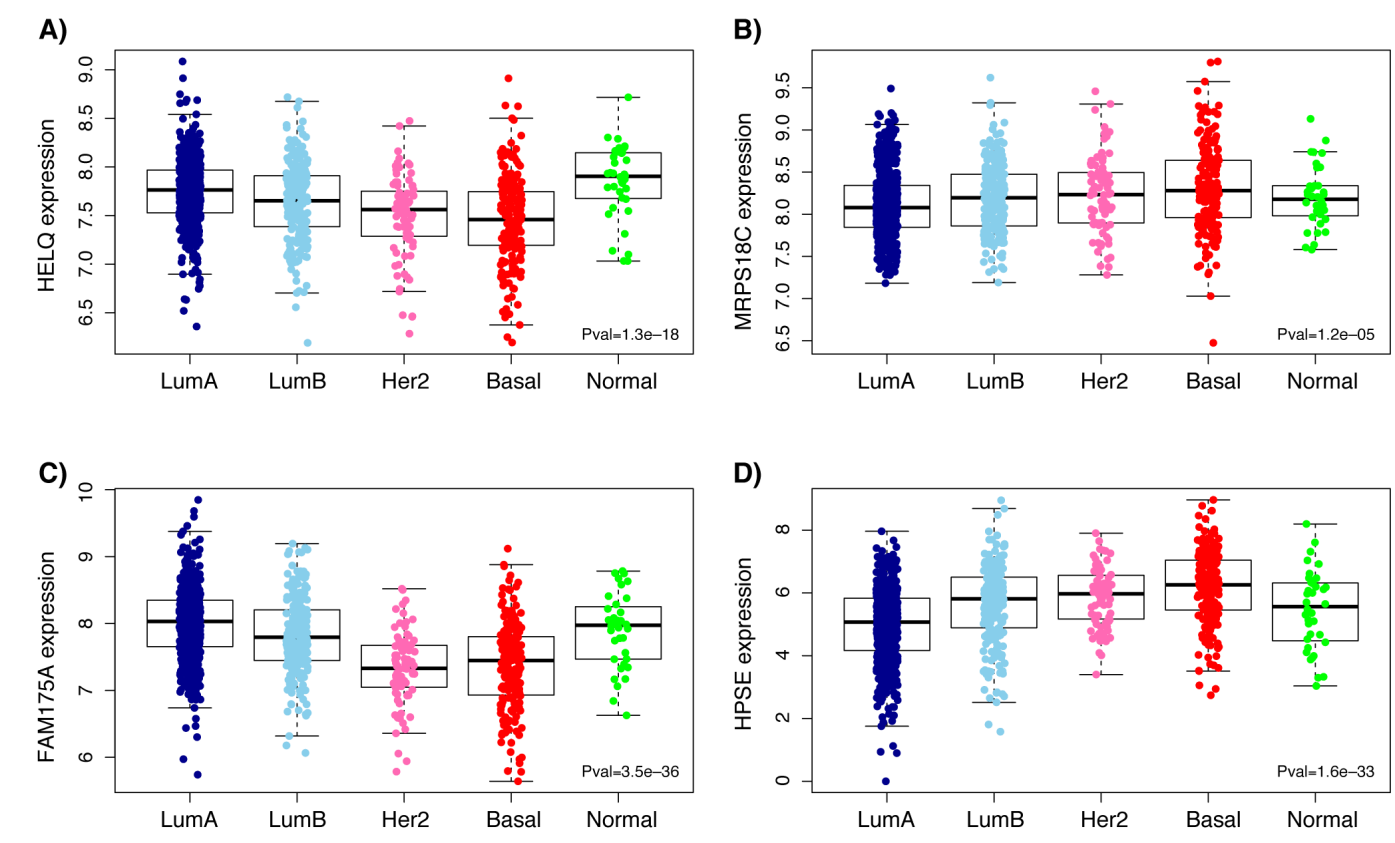
Boxplots representing expression levels of *HELQ (A), MRPS18C (B), FAM175A (C)* and *HPSE (D)* in the 5 molecular subtypes (PAM50 classifier) of breast primary tumors Differential expression between normal breast and tumor tissue was determined by a Kruskal-Wallis rank sum test. Analysis was performed using TCGA breast cancer RNAseq data from five molecular subtypes of breast primary tumors: Luminal A (LumA), Luminal B (LumB), Human epidermal growth factor receptor 2-enriched (Her2), Basal-like (Basal) and Normal-like (Normal). Horizontal bars indicate mean expression levels.

Analysis of RNASeq data from ENCODE showed high levels of expression for *MRPS18C* in both HMEC and MCF-7 while *HELQ* and *FAM175A* are expressed at very low levels in these cell lines (Figure 2D). However, as illustrated in Figure 3, analysis of TCGA breast cancer RNAseq data in primary tumor (*n* = 765), adjacent normal (*n* = 93) and metastasis (*n* = 6) showed that *HELQ*, *FAM175A* and *HPSE*, but not *MRPS18C*, were all found to be differentially expressed between normal breast and tumor tissue (*P* = 1x10^-45^, *P* = 6.6x10^-31^, *P* = 7.3x10^-10^, and *P* = 0.28, respectively, as determined by a Kruskal-Wallis rank sum test). Further analysis comparing the tumor expression levels of these genes between the 5 molecular subtypes of breast cancer, namely: Luminal A, Luminal B, Her2-enriched, Basal-like and Normal-like, showed that while *HELQ* and *FAM175A* expression levels are decreased in Basal-like tumors (*P* = 1.3x10^-18^ and *P* = 3.5x10^-36^, respectively (Kruskal-Wallis test), *MRPS18C* and *HPSE* expression were found to be up regulated in Basal-like carcinomas (*P* = 1.2x10^-5^, *P* = 1.6x10^-33^) (Figure 4).

### Expression quantitative trait locus analysis (eQTL) in breast tissue

**Figure 5:**
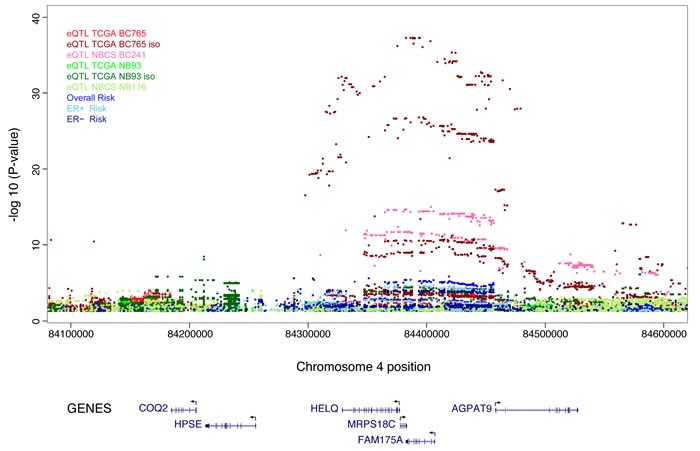
Manhattan plots of association for the eQTL results at the 4q21 locus in normal breast and breast cancer tissue *Y*-axis shows -log10(*P*-value) while *x*-axis shows physical position. Circles of various shades of blue represent breast cancer risk associations for all breast cancer tumors, ER+ and ER- tumors. Other colored circles represent eQTL results in the following datasets: normal breast (NB93, NB116) in various shades of green, breast carcinomas in pink (BC241) and red (BC765). Risk association results as well as eQTL results are for both imputed and genotyped SNPs for all datasets.

**Figure 6:**
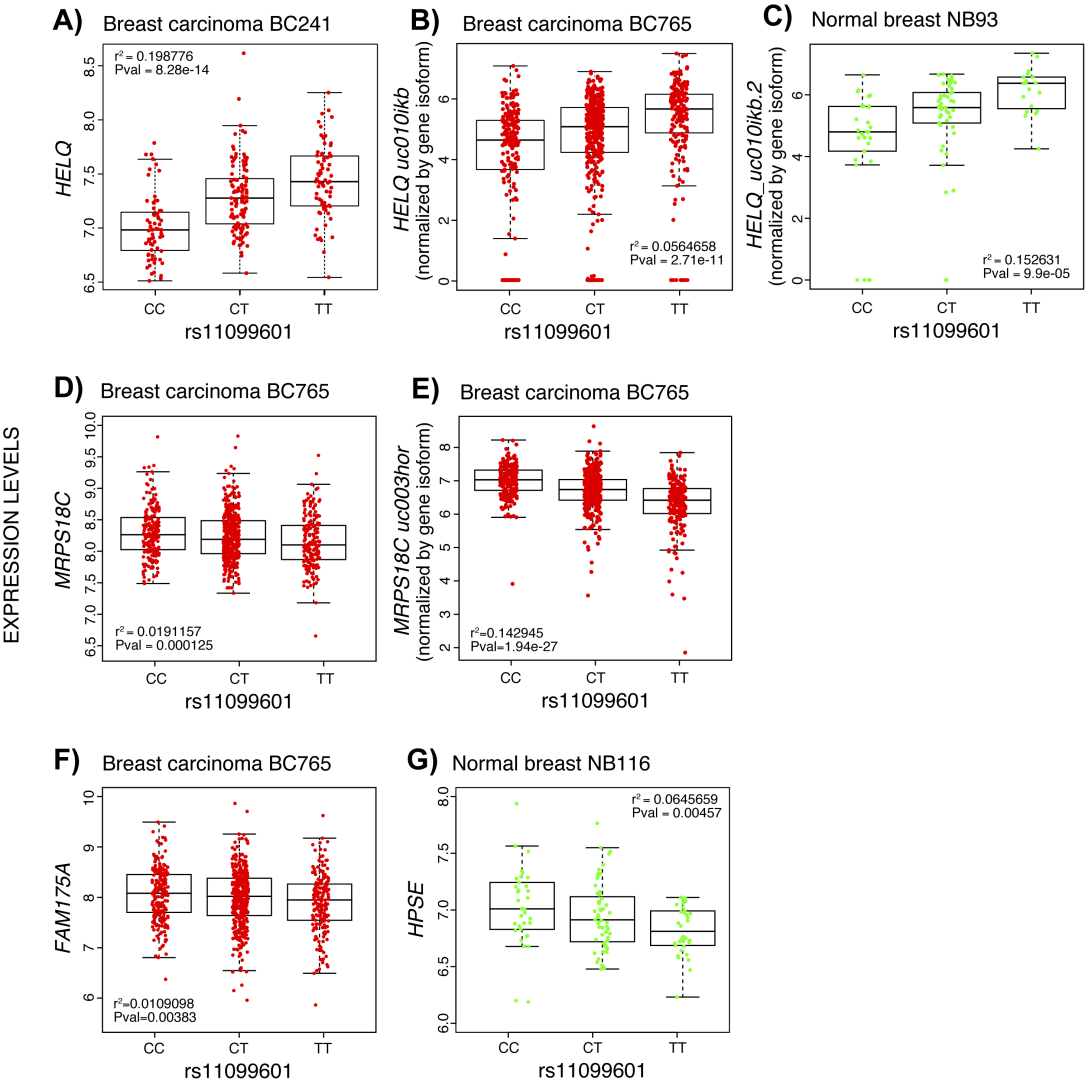
Boxplots representing the most significant eQTL results for variant rs11099601 in normal breast tissue and breast tumor datasets Box plots represent the expression levels of the indicated transcripts with respect to the rs11099601 genotypes. Expression levels are shown for **A.** *HELQ* in breast carcinoma BC241 dataset, **B.** *HELQ* in breast carcinoma BC765 dataset normalized per isoform, **C.** *HELQ* in normal breast NB93 dataset normalized by gene isoform, **D.** *MRPS18C* in breast carcinoma BC765 dataset, **E.** *MRPS18C* in breast carcinoma BC765 dataset normalized per isoform, **F.** *FAM175A* in breast carcinoma BC765 dataset and **G.** *HSPE* in normal breast NB116 dataset. Horizontal bars indicate mean expression level per genotype. r^2^ values indicate the percentage of variance in respective gene expression levels explained by rs11099601.

In order to identify associations between candidate variants and expression levels of genes within the 4q21 region, we analyzed all genotyped and imputed SNPs within a 1Mb region centered around the most significant SNP (rs11099601), in normal and breast cancer tissue. Significant eQTL associations were observed for numerous SNPs in the region in both normal breast and tumors (Figure 5). In the breast cancer tissue dataset BC241, the most strongly expression-associated SNP at this locus was our top risk SNP rs11099601, which was associated with expression levels of *HELQ*, (with *P* = 8.28x10^-14^ and r^2^ = 0.20, where the r^2^ value indicates the percentage of variance in *HELQ* expression levels explained by rs11099601) (Figure 6A). A decrease in *HELQ* expression levels was observed with increasing copy number of the rs11099601 (C) allele (Figure 6A). Multiple SNPs within the 1 Mb region were also associated with expression of *HELQ*, all of which were correlated with rs11099601 (r^2^ > 0.3). No significant eQTLs were observed between rs11099601 and other genes in this region, namely *COQ2*, *HPSE*, *MRPS18C*, *FAM175A*, or *AGPAT9*, using data from the BC241 sample set.

In the TCGA BC765 breast cancer dataset, *HELQ* expression levels were not associated with rs11099601 (*P* = 0.34 and r^2^ = 0.00099) or with any other SNPs in this region. Weak associations were only observed between rs11099601 and expression levels for *MRPS18C* (*P* = 1.25x10^-4^ and r^2^ = 0.02) (Figure 6D) and *FAM175A* (*P* = 3.83x10^-3^ and r^2^ = 0.011) (Figure 6F).

Further isoform-specific analysis was performed in the TCGA BC765 breast cancer dataset. In contrast to the expression data generated from the Norwegian sample sets, which were obtained using expression arrays, expression data from the TCGA datasets used in the current study were obtained by RNA-Sequencing, thus allowing further analysis of different gene isoforms. Thus, in the BC765 dataset, these analyses resulted in the identification of significant eQTLs for an isoform of *HELQ* (uc101ikb) (*P* = 2.71x10^-11^ and r^2^ = 0.056) (Figure 6B), corresponding to a long isoform of the gene with one exon lacking. These analyses also further revealed highly significant associations for the *MRPS18C* isoform uc003hor (*P* = 1.94x10^-27^ and r^2^ = 0.143) (Figure 6E).

Similar to what is observed in the TCGA BC765 breast cancer dataset, gene-normalized analysis in the TCGA normal breast tissue dataset NB93 did not reveal associations between *HELQ* expression levels and rs11099601 while isoform-normalized analysis showed associations with *HELQ* isoform uc101ikb (*P* = 9.90x10^-05^ and r^2^ = 0.153) (Figure 6C).

In normal breast tissue from the NBCS (NB116), the strongest eQTLs were observed for *HPSE*, where rs11099601 was associated with a decrease in *HPSE* expression levels (*P =* 4.57x10^-3^, r^2^ = 0.0645) (Figure 6G). rs11099601 was not associated with the expression levels of any other genes in this region.

Although associations were detected between several genes and our top risk SNP in the different sample sets, a lack of consistency in eQTL associations between the two breast cancer sample sets was observed. It should be noted that expression data were obtained trough different approaches as previously mentioned, i.e expression array (44K Agilent array) for BC241 and RNA-Sequencing for BC765 (Illumina RNAseq). Moreover, there are differences in the overall PAM50 subtype distributions between these two sample sets. As depicted in S3 Figure, differences are noted mainly in the distribution of Luminal A (28.22% in BC241 compared to 49.33% for BC765), Her2 (15.35% in BC241 compared to 8.16% for BC765) and Normal-like (14.52% in BC241 compared to 2.41% for BC765) subtypes. Expression levels of *HELQ*, and other candidate genes, were shown to vary significantly between these molecular subtypes (Figure 4) and thus a different distribution of these subtypes between the two sample sets could explain the underlying lack of replication in the eQTL analyses.

## DISCUSSION

It is well recognized that genetic variants located in genomic regions that regulate gene expression are major causes of human diversity and may also be important susceptibility factors for complex diseases and traits. Indeed, it has been shown that approximately 30% of expressed genes show evidence of *cis*-regulation by common polymorphic alleles [47]. Moreover, in recent years, GWAS have identified thousands of variants associated with various diseases/traits, ~90% of which localize outside of known protein-coding regions [42, 43], implicating a regulatory role for these variants.

In the present study, we have assessed the association with breast cancer risk of 313 regulatory SNPs in genes involved in the etiology of cancer (see S1 Table for complete list of SNPs and genes), in 46,451 breast cancer cases and 42,599 controls of European ancestry. Using this approach, we identified rs11099601 (OR = 1.05, *P* = 5.6x10^-6^), a novel breast cancer susceptibility locus on chromosome 4q21. Analysis of imputed SNPs across a 500Kb region surrounding rs11099601 revealed that this variant remained one of the strongest risk signals, tagging a set of 76 strongly correlated SNPs across a 135Kb LD block containing several genes, including *COQ2, HPSE, HELQ, MRPS18C, FAM175A (ABRAXAS)* and *AGPAT9*.

Functional annotation of the 4q21 locus with ENCODE biofeatures in mammary cell lines pointed toward rs11099601 as one of the most likely functional variants in this region. eQTL analysis showed significant eQTLs in normal and breast cancer tissue for several variants in the 4q21 region, including rs11099601. The strongest associations for rs11099601 and expression were observed in breast carcinomas for *MRPS18C* and *HELQ* and explain approximately 14% and 20% of their expression variance, respectively (Figure 6). Other genes whose expression correlated with this eQTL included *HPSE* and *FAM175A*.

These genes represent interesting candidates for further analyses related to breast cancer susceptibility. Indeed, analysis of TCGA breast cancer RNAseq data showed that *HELQ*, *FAM175A* and *HPSE* were found to be differentially expressed between normal breast and tumor tissue and further analysis showed that *HELQ* and *FAM175A* expression levels are significantly decreased in basal-like tumors.

*HELQ* is a single-stranded DNA-dependent ATPase and DNA helicase involved in DNA repair and signaling in response to ICL. Genetic disruption of *HELQ* in human cells enhances cellular sensitivity and chromosome radial formation by the ICL-inducing agent mitomycin C (MMC). After treatment with MMC, reduced phosphorylation of CHK1 occurs in knockout cells and accumulation of G2/M cells is reduced [51]. Furthermore, it was recently shown that Helq helicase-deficient mice exhibit subfertility, germ cell attrition, ICL sensitivity, and tumor predisposition [52]. A meta-analysis of 22 GWAS, as well as a recent GWAS involving ~70,000 women performed in the BCAC, have both identified rs4693089, located in an intron of *HELQ* and perfectly correlated with rs11099601, as associated with age at natural menopause (p = 2.4x10^-19^ and p = 9.2x10^-23^, respectively) [53, 54]. Moreover, a GWAS of upper aero-digestive tract cancers conducted by the International Head and Neck Cancer Epidemiology Consortium identified rs1494961, a missense mutation V306I in the second exon of *HELQ* gene perfectly correlated with rs11099601 (r^2^ = 1), to be associated with increased risk of upper aero-digestive tract cancers in their combined analysis (*P* = 1x10^-8^) [55]. Another study by the same group analyzed the role of DNA repair pathways in upper aero-digestive tract cancers [56]. This study showed that the polymerase pathway, to which the *HELQ* gene belongs, is the only pathway significant for all upper aero-digestive tract cancer sites combined and that this association is entirely explained by the association with rs1494961 (*P* = 2.65×10^-4^) [56]. Finally, a recent study reported the mutation screening of *HELQ* in 185 Finnish breast or ovarian cancer families [57]. This study did not provide evidence for a role of *HELQ* in breast cancer susceptibility in the Finnish population, but analyses in other populations and larger datasets are needed to further assess its role in breast cancer predisposition [57], especially with regard to the involvement of rare variants. In the current study, we have shown *HELQ* to be differentially expressed between normal breast and tumor tissue and to be significantly down regulated in basal-like breast tumors compared to ER positive tumors, suggesting that altered gene expression levels, potentially mediated through the effect of regulatory variants, could be one of the mechanisms contributing to breast cancer susceptibility. Previous studies have provided some evidence, in known breast cancer susceptibility genes *BRCA1* [58] and *BRCA2* [59], of genetic variants associated with allelic expression differences which could affect the risk of breast cancer in mutation carriers through altering expression levels of the wild-type allele. Also, a recent study showed suggestive associations between DAE associated variants located in breast cancer susceptibility chromosomal regions, and prognosis (*ZNF331* and *CHRAC1*) [60].

Another gene in this locus, *FAM175A*, is involved in DNA damage response and double-strand break (DSB) repair. It is a component of the BRCA1-A complex, acting as a central scaffold protein that assembles the various components of the complex and mediates the recruitment of BRCA1 [61-63]. Further evidence rendering *FAM175A/ABRAXAS* an interesting candidate gene is a recent report showing that both homozygous and heterozygous *Abraxas* knockout mice exhibited decreased survival and increased tumor incidence [64]. This study also showed that somatic deletion of the *ABRAXAS* locus on chromosome 4q21 is found in human ovarian and breast cancers (especially basal subtype), and this loss is well correlated with reduced *ABRAXAS* expression in these cancers [64]. Moreover, Solyom et al. reported a novel germline *ABRAXAS* mutation (p.Arg361Gln) in Northern Finnish breast cancer families which affects the nuclear localization of the protein and consequently reduces the formation of BRCA1 and Rap80 foci at DNA damage sites, leading to ionizing radiation hypersensitivity of cells and partially impairing the G2/M checkpoint [65]. Our group has also, in parallel to the present study, conducted a population-based case-control mutation screening study of the coding exons and exon/intron boundaries of *ABRAXAS* in 1250 breast cancer cases and 1250 controls from the Breast Cancer Family Registry, including individuals from different ethnic groups such as Caucasian, Latino, East Asian and African-American ancestry. Although this study did not reveal evidence of association of the identified variants with breast cancer risk, two variants were identified and were shown to diminish the phosphorylation of γ-H2AX, an important biomarker of DNA double-strand breaks [66].

Lastly, *MRPS18C* encodes a protein that belongs to the ribosomal protein S18P family, which includes three proteins (MRPS18A, MRPS18B, MRPS18C) having significant sequence similarity to bacterial S18 proteins. MRPS18C is part of the small subunit (28S) of the mitochondrial ribosome involved in oxidative phosphorylation and thus the role of this protein in breast cancer susceptibility is unclear. It was reported that MRPS18B (MRPS18-2) binds to RB [67] and prevents the formation of the E2F1-RB complex that leads to elevated levels of free E2F1 protein in the nucleus and the subsequent promotion of S phase entry [68]. Overexpression of human MRPS18B caused transformation of terminally differentiated rat skin fibroblasts and transformed cells became tumorigenic in SCID (severe combined immunodeficiency) mice [69]. These transformed cells showed anchorage-independent growth and loss of contact inhibition; they expressed epithelial markers, showed increased telomerase activity, disturbance of the cell cycle, and chromosomal instability, leading the authors to suggest that MRPS18B is a newly identified oncoprotein [69]. Although these results suggest that MRPS18B may be involved in carcinogenesis, there is currently no evidence showing that MRPS18C is involved in processes other than oxidative phosphorylation.

## CONCLUSION

Phenotypic differences among cell types, individuals, and populations are determined by variation in gene expression, a substantial proportion of which is driven by genetic variants residing in regulatory elements near the affected genes. Analysis of variants associated with differential allelic expression has allowed us to identify a novel locus on chromosome 4q21 associated with breast cancer risk. Subsequent tissue specific eQTL analyses have confirmed significant eQTLs for this locus in both normal and breast cancer tissue.

At the time of study design, data on differential allelic expression was not available in breast tissue, leading us to perform the selection of candidate variants in other cell types such as lymphoblastoid cell lines, fibroblasts and monocytes. This constitutes a limitation of our study which may explain why some of the associations observed between the selected variants and DAE in these cells types were not replicated in the eQTL analyses performed in normal breast and/or breast cancer cells. Indeed, SNPs associated with variation in gene expression have now been mapped for a variety of tissues, highlighting their tissue dependent properties and the need for expression profiling of a diverse panel of cell types.

Hence, further functional characterization of the 4q21 locus, and replication in a larger dataset, would be relevant to provide more robust evidence of the involvement of this region in breast cancer susceptibility as well as identify the gene(s) and biological mechanism(s) underlying this susceptibility.

## MATERIALS AND METHODS

### Sample selection

A total of 46,451 breast cancer cases and 42,599 controls of European ancestry were included from 41 studies participating in the Breast Cancer Association Consortium (BCAC). Studies were population-based or hospital-based case-control studies, including nested case-control studies within cohorts. Some studies selected cases by age, or oversampled cases with a family history (S5 Table). Studies provided ~2% of samples in duplicate for quality control purposes (see below). Study subjects were recruited on protocols approved by the Institutional Review Boards at each participating institution, and all subjects provided written informed consent.

### SNP selection

SNP selection was performed by first identifying a list of genes of interest, which was determined by the involvement of these genes in cancer related pathways and/or mechanisms. The list of genes was established by researching published results and/or by using available public databases such as the Kyoto Encyclopedia of Genes and Genomes (KEGG) (http://www.genome.jp/kegg/). Thereafter, DEA SNPs falling within these gene regions were identified using previously reported data on allelic expression *cis*-associations, derived using: 1) the llumina Human1M-duo BeadChip for lymphoblastoid cell lines from Caucasians (CEU population) (*n* = 53) [47], the Illumina Human 1M Omni-quad for primary skin fibroblasts derived from Caucasian donors (*n* = 62) [49, 70], and the Illumina Infinium II assay with Human 1.2 M Duo custom BeadChips v1 for human primary monocytes (*n* = 188) [71]. Briefly, 1000 Genomes project data was used as a reference set (release 1000G Phase I v3) for the imputation of genotypes from HapMap individuals. Untyped markers were inferred using algorithms implemented in IMPUTE2. The unrelated fibroblast panel consisted of 31 parent-offspring trios, where the genotypes of offspring were used to allow for accurate phasing. Mapping of each allelic expression trait was carried out by first normalizing allelic expression ratios at each SNP using a polynomial method [72] and then calculating averaged phased allelic expression scores across annotated transcripts, followed by correlation of these scores to local (transcript +/-500 kb) SNP genotypes in fibroblasts as described earlier [70].

Three hundred fifty-five genetic variants were selected on the basis of evidence of association with DAE in 175 genes involved in cancer-related pathways as described above (see S1 Table for complete list of SNPs and genes). Following selection, SNPs were submitted for design and inclusion on a custom Illumina Infinium array (iCOGS), as part of a BCAC genotyping initiative (see Genotyping and Quality Control section below). After undergoing design and post-genotyping quality control, 313 SNPs remained for analysis.

### Genotyping and quality control

Genotyping was carried out as part of a collaboration between BCAC and three other consortia (the Collaborative Oncological Gene-environment Study, COGS). Full details of SNP selection, array design, genotyping and post-genotyping quality control (QC) have been published [35]. Briefly, three categories of SNPs were chosen for inclusion on the array: (i) SNPs selected on the basis of pooled GWAS data, (ii) SNPs selected for the fine-mapping of published risk loci and (iii) candidate SNPs selected on the basis of previous analyses or specific hypotheses. The 313 SNPs described in the current study were candidate SNPs selected on the basis of the hypothesis that regulatory variants are involved in breast cancer susceptibility. In general, only SNPs with an Illumina design score of 0.8 or greater were considered. SNPs were preferentially accepted if they had a design score of 1.1 (i.e. had previously been genotyped on an Illumina platform). If not, we sought SNPs with r^2^ = 1 with the selected SNP, and selected the SNP with the best design score. If no such SNP was available, we selected SNPs with r^2^ > 0.8 with the chosen SNP, and selected the SNP with the best design score. For the COGS project overall, genotyping of 211,155 SNPs in samples was conducted using a custom Illumina Infinium array (iCOGS) in four centers. Genotypes were called using Illumina's proprietary GenCall algorithm. Standard quality control measures were applied across all SNPs and all samples genotyped as part of the COGS project [35]. After quality control, genotype data were available for 48 155 breast cancer cases and 43 612 controls, and call rates for all SNPs were > 95%.

### Statistical analysis

Per-allele log-odds ratios (ORs) were estimated using logistic regression, adjusted for principal components and study, as described previously [35]. *P*-values were estimated using Wald test. For imputation, genotype data from 48,155 breast cancer cases and 43,612 controls were used to estimate genotypes for other common variants across a 500 kb region on chromosome 4 (chr4: 84,132,763-84,632,763 - NCBI build 37 assembly), with IMPUTE v.2.2 and the March 2012 release of the 1,000 Genomes Project as reference panel. In all analyses, only SNPs with imputation information/accuracy r2 > 0.30 were considered [40].

### Linkage disequilibrium

LD values were computed using 118 independent individuals from the CEU population of the 1,000 Genome dataset (v3, release 20110521, downloaded from 1000genomes.ebi.ac.uk on April 2013) [73]. The relevant subset was extracted from the raw data using VCFtools (v0.1.7) [74] and the paired r^2^ statistics were obtained for all target loci using PLINK! (v1.07) [75]. The linkage heatmaps and the association plots were produced on the R platform (v3.0) using the package LDheatmap [76].

### Breast cancer association analyses performed in *BRCA1* and *BRCA2* mutation carriers

Associations with breast cancer risk were evaluated within a retrospective cohort framework, by modelling the retrospective likelihood of the observed genotypes conditional on the disease phenotype. These analyses are described in detail elsewhere [77, 78].

### Functional annotation

Two publicly available tools, RegulomeDB [79] and HaploReg V4 [80], were also used to evaluate candidate variants. For a full description of the RegulomeDB scoring scheme refer to (http://www.regulomedb.org).

Publicly available genomic data was also used to annotate each SNP most strongly associated with breast cancer risk at locus 4q21 (for data sources refer to S6 Table). The following regulatory features were obtained for breast cell types from ENCODE and NIH Roadmap Epigenomics data through the UCSC Genome Browser: DNase I hypersensitivity sites, Chromatin Hidden Markov Modelling (ChromHMM) states, histone modifications of epigenetic markers more specifically commonly used marks associated with enhancers (H3K4Me1 and H3K27Ac) and promoters (H3K4Me3 and H3K9Ac), and transcription factor ChiP-seq data.

To identify putative target genes, we examined potential functional chromatin interactions between distal and proximal regulatory transcription-factor binding sites and the promoters at the risk loci, using the Chromatin Interaction Analysis by Paired End Tag (ChiA-PET) and Genome conformation capture (Hi-C, 3C and 5C) datasets downloaded from GEO (for data sources refer to S6 Table).

Maps of active mammary super-enhancer regions in HMEC cells were obtained from Hnisz et al. [81]. Predicted enhancer-promoter determined interactions were obtained from the integrated method for predicting enhancer targets (IM-PET) described in He et al. [82].

RNA-Seq data from ENCODE was used to evaluate the expression of exons across the 4q21 locus in HMEC and MCF7 cell lines. For HMEC and MCF7, alignment files from 4 and 19 expression datasets respectively were downloaded from ENCODE using a rest API wrapper (ENCODExplorer R package) [83] in the bam format and processed using metagene R packages [84] to normalize in Reads per Millions aligned, and to convert in coverages.

### eQTL analyses

The influence of germline genetic variations on gene expression was assessed using a linear regression model, as implemented in the R library eMAP (http://www.bios.unc.edu/~weisun/software.htm). An additive effect was assumed by modeling subjects’ copy number of the rare allele, i.e. 0, 1 or 2 for a given genotype. Only relationships in *cis* (defined as those in which the SNP resided less than 1 MB up or down from the center of the transcript) were investigated. eQTL analyses were performed on both normal breast and tumor tissues, and included the following materials: Normal Breast: NB116 (*n* = 116) consists of samples from women of Caucasian ancestry recruited in Oslo, comprising expression data from normal breast biopsies (*n* = 73), reduction plastic surgery (*n* = 34) and adjacent normal (*n* = 9) (adjacent to tumour). Genotyping was performed using the iCOGS SNP array, and gene expression levels were measured with Agilent 44K [85, 86]. NB93 is the Caucasian fraction of the TCGA dataset for which adjacent normal breast expression data were available, *n* = 93 for the data normalized per gene, and *n* = 94 for the data normalized per isoform. Birdseed processed germline genotype data from the Affy6 SNP array were obtained from the TCGA dbGAP data portal [87]. Gene expression levels were assayed by RNA sequencing, RSEM (RNAseq by Expectation-Maximization, [88] normalized both per gene and per isoform, as obtained from the TCGA consortium [87]. The data was log2 transformed, and unexpressed genes were excluded prior to eQTL analysis. Breast carcinomas: BC241, is a Caucasian sample set recruited from Oslo, *n* = 241. The sample set includes all stages of breast cancer, and genotypes were obtained with the iCOGS SNP array, and mRNA expression data was from the Agilent 44K array [86, 89]. BC765 comprises samples from the TCGA breast cancer sample set of Caucasian origin [87], *n* = 765 for the data normalized per gene, and *n* = 766 for the data normalized per isoform. Genotyping platform was Affy6, and gene expression was measured using RNA sequencing. See NB93 for a more detailed description. For all sample sets, the genotyping data was processed as follows: SNPs with call rates < 0.95 or minor allele frequencies < 0.05 were excluded, as were SNPs out of Hardy Weinberg equilibrium with *P* < 10^-13^. All samples with a call rate below 80% were excluded. Identity by state was computed using the R GenABEL package [90], and closely related samples with IBS > 0.95 were removed. The SNP and sample filtration criteria were applied iteratively until all samples and SNPs met the stated thresholds.

## SUPPLEMENTARY MATERIALS FIGURES AND TABLES














