# Supplementary material for: Association of breast cancer risk with genetic variants showing differential allelic expression: Identification of a novel breast cancer susceptibility locus at 4q21
Source: Oncotarget. 2016 Oct 22;7(49):80140–63. doi: 10.18632/oncotarget.12818 (PMC5340257; doi:10.18632/oncotarget.12818)
Supplement: Supplementary file 1 [file oncotarget-07-80140-s001.pdf]

Association of breast cancer risk with genetic variants showing differential allelic expression: Identification of a novel breast cancer susceptibility locus at 4q21

Supplementary Material

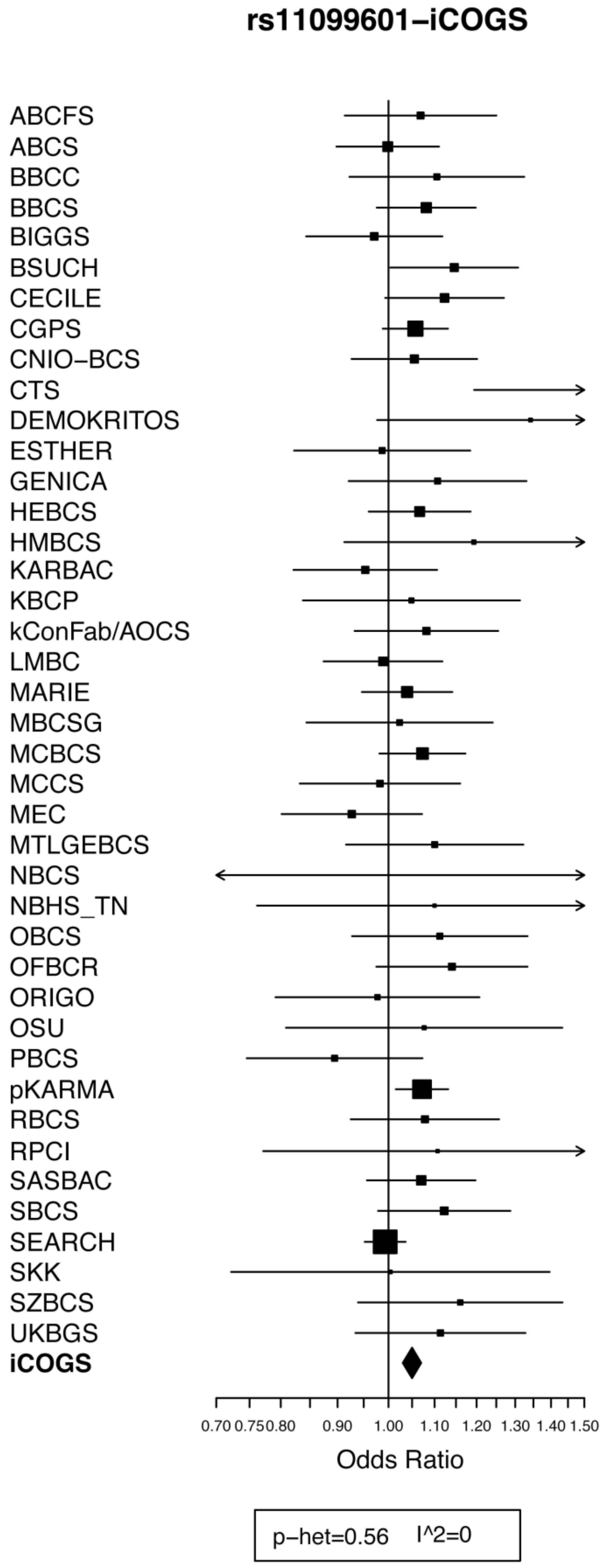

rs11099601−iCOGS
ABCFS
ABCS
BBCC
BBCS
BIGGS
BSUCH
CECILE
CGPS
CNIO−BCS
CTS
DEMOKRITOS
ESTHER
GENICA
HEBCS
HMBCS
KARBAC
KBCP
kConFab/AOCS
LMBC
MARIE
MBCSG
MCBCS
MCCS
MEC
MTLGEBCS
NBCS
NBHS_TN
OBCS
OFBCR
ORIGO
OSU
PBCS
pKARMA
RBCS
RPCI
SASBAC
SBCS
SEARCH
SKK
SZBCS
UKBGS
iCOGS
0.70 0.75 0.80 0.90 1.00 1.10 1.20 1.30 1.40 1.50
Odds Ratio
p−het=0.56 I^2=0

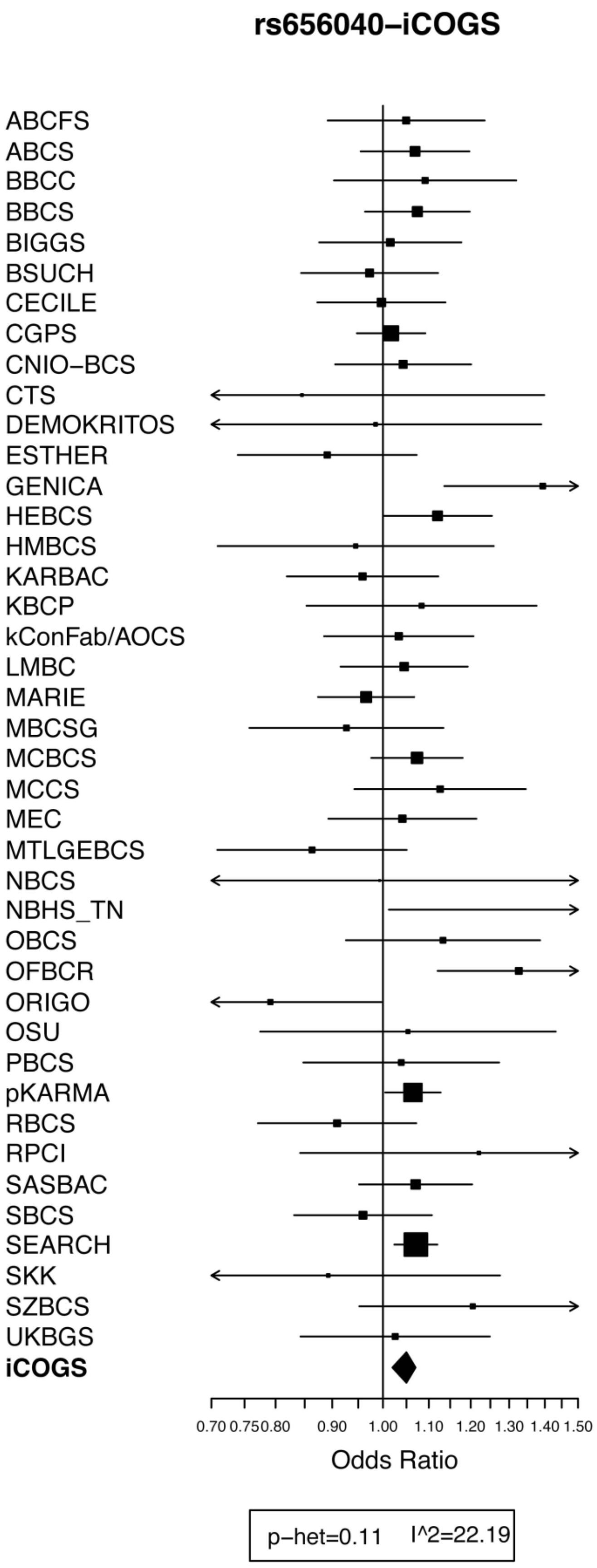
rs656040−iCOGS
ABCFS
ABCS
BBCC
BBCS
BIGGS
BSUCH
CECILE
CGPS
CNIO−BCS
CTS
DEMOKRITOS
ESTHER
GENICA
HEBCS
HMBCS
KARBAC
KBCP
kConFab/AOCS
LMBC
MARIE
MBCSG
MCBCS
MCCS
MEC
MTLGEBCS
NBCS
NBHS_TN
OBCS
OFBCR
ORIGO
OSU
PBCS
pKARMA
RBCS
RPCI
SASBAC
SBCS
SEARCH
SKK
SZBCS
UKBGS
iCOGS
0.70 0.75 0.80 0.90 1.00 1.10 1.20 1.30 1.40 1.50
Odds Ratio
p−het=0.11 I^2=22.19

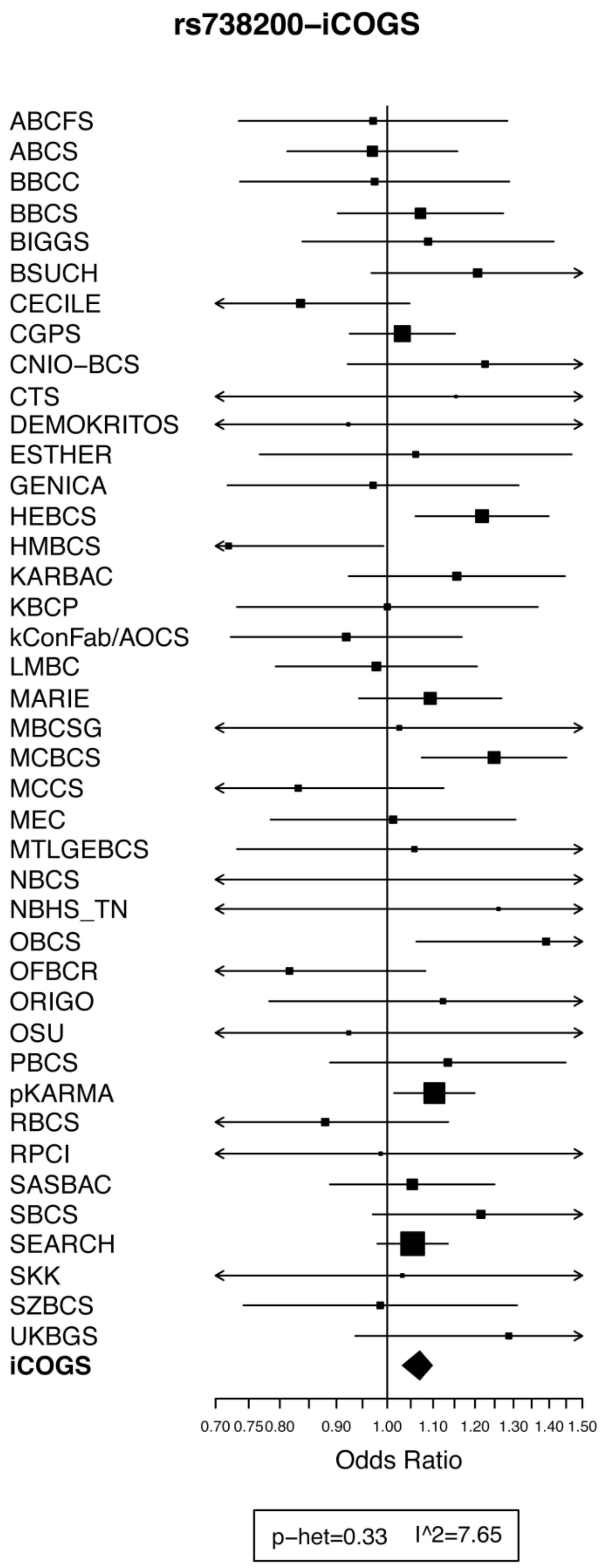
rs738200–iCOGS
ABCFS
ABCS
BBCC
BBCS
BIGGS
BSUCH
CECILE
CGPS
CNIO–BCS
CTS
DEMOKRITOS
ESTHER
GENICA
HEBCS
HMBCS
KARBAC
KBCP
kConFab/AOCS
LMBC
MARIE
MBCSG
MCBCS
MCCS
MEC
MTLGEBCS
NBCS
NBHS_TN
OBCS
OFBCR
ORIGO
OSU
PBCS
pKARMA
RBCS
RPCI
SASBAC
SBCS
SEARCH
SKK
SZBCS
UKBGS
iCOGS
0.70 0.75 0.80 0.90 1.00 1.10 1.20 1.30 1.40 1.50
Odds Ratio
p–het=0.33
I^2=7.65

S1 Fig. Forest plots for the three most significant SNPs (overall *P*-value <10^{-4}). Squares indicate the estimated per-allele OR for the minor allele in Europeans. The horizontal lines indicate 95% confidence limits. The area of the square is inversely proportional to the variance of the estimate. The diamond indicates the estimated per-allele OR from the combined analysis.

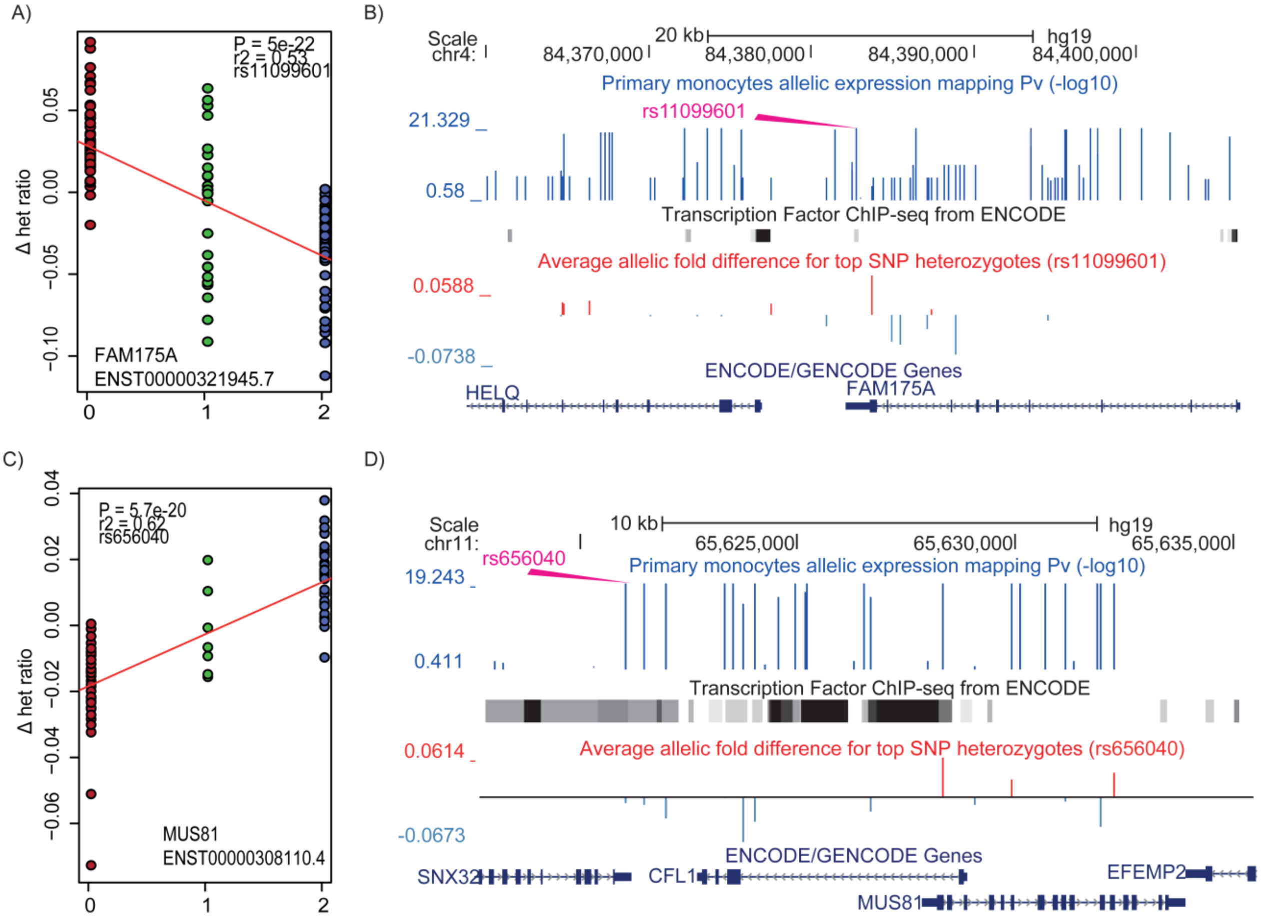

S2 Fig. Differential allelic expression mapping of *FAM175A* and *MUS81* loci. (A) (C) The most significant *cis*-regulatory variants mapped by regression analysis in the primary monocyte population for *FAM175A* and *MUS81* are rs11099601 ($P=5\times10^{-22}$) (A) and rs656040 ($P=5.7\times10^{-20}$) respectively (C). Screenshot of the rs11099601 (B) and rs656040 (D) regions from the UCSC genome browser. Tracks display from top to bottom the *P*-values (-log10) of the allelic expression mapping in primary monocytes for each SNP, transcription factor binding (ENCODE

ChIP-seq data) and average allelic expression across all individuals heterozygous for rs11099601 (B) and rs656040 (D).

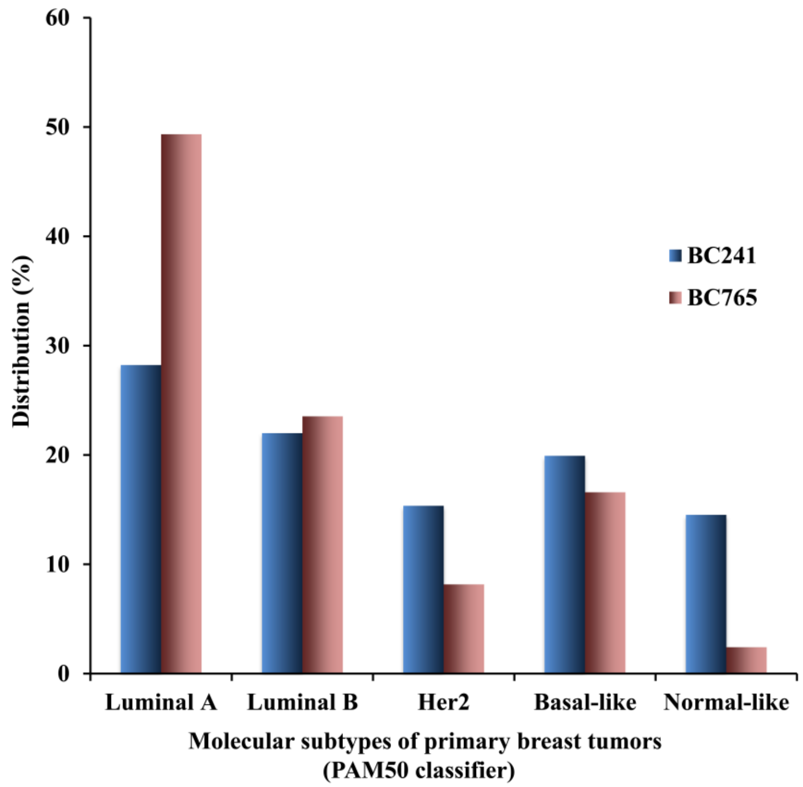

S3 Fig. Distribution of the 5 molecular subtypes (PAM50 classifier) of breast primary tumors in the two breast cancer samples sets used for eQTL analysis – BC241 and BC765. The distribution of Luminal A, Luminal B, Human epidermal growth factor receptor 2-enriched (Her2), Basal-like and Normal-like subtypes is represented as the percentage of the total number of samples in each sample set.

S1 Table. List of selected genes and genetic variants associated with differential allelic expression.

S2 Table. Associations for the 313 genotyped SNPs with overall, ER-positive and ER-negative breast cancer risk.

S3 Table. **Associations for imputed and genotyped SNPs in the 4q21 locus (4q21: 84,132,874-84,631,193) for overall, ER-positive and ER-negative breast cancer risk.**

S4 Table. Regulome DB analysis of SNPs in the 4q21 locus (4q21: 84,132,874-84,631,193) with r^2>0.8 with top associated SNP rs11099601. The scoring scheme refers to the following available datatypes: **1a** = eQTL + transcription factor (TF) binding + matched TF motif + matched DNase Footprint + DNase peak; **1b** = eQTL + TF binding + any motif + DNase Footprint + DNase peak; **1c** = eQTL + TF binding + matched TF motif + DNase peak; **1d** = eQTL + TF binding + any motif + DNase peak; **1e** = eQTL + TF binding + matched TF motif; **1f** = eQTL + TF binding / DNase peak; **2a** = TF binding + matched TF motif + matched DNase Footprint + DNase peak; **2b** = TF binding + any motif + DNase Footprint + DNase peak; **2c** = TF binding + matched TF motif + DNase peak; **3a** = TF binding + any motif + DNase peak; **3b** = TF binding + matched TF motif; **4** = TF binding + DNase peak; **5** = TF binding or DNase peak; **6** = other.

S5 Table. Description of the BCAC studies with subjects of European origin contributing to iCOGs.

S6 Table. Data sources for *in silico* analyses of the 4q21 breast cancer susceptibility loci
